# Short-term elevation of intracranial pressure does neither influence duodenal motility nor frequency of bolus transport events: a porcine model

**DOI:** 10.1186/1471-227X-6-1

**Published:** 2006-01-25

**Authors:** Joerg Schnoor, Norbert Zoremba, Marcus C Korinth, Bjoern Kochs, Jiri Silny, Rolf Rossaint

**Affiliations:** 1 Department of Anaesthesiology, University Hospital Aachen, Germany; 2 Department of Neurosurgery, University Hospital Aachen, Germany; 3 Femu-Research Institute, University Hospital Aachen, Germany

## Abstract

**Background:**

Patients with traumatic brain injuries and raised intracranial pressure (ICP) display biphasic response with faster gastric emptying during the early stage followed by a prolonged gastric transit time later. While duodenal contractile activity plays a pivotal role in transpyloric transit we investigated the effects of raised intracranial pressure on duodenal motility during the early phase. In order to exclude significant deterioration of mucosal blood supply which might also influence duodenal motility, luminal microdialysis was used in conjunction.

**Methods:**

During general anaesthesia, 11 pigs (32–37 kg, German Landrace) were instrumented with both a luminal catheter for impedancometry and a luminal catheter for microdialysis into the proximal duodenum. Additionally, a catheter was inserted into the left ventricle to increase the intracranial pressure from baseline up to 50 mmHg in steps of 10 mmHg each hour by injection of artificial cerebrospinal fluid. At the same time, duodenal motility was recorded continuously. Duodenal luminal lactate, pyruvate, and glucose concentrations were measured during physiological state and during elevated intracranial pressure of 10, 20, 30, 40, and 50 mmHg in six pigs. Five pigs served as controls.

**Results:**

Although there was a trend towards shortened migrating motor cycle (MMC) length in pigs with raised ICP, the interdigestive phase I–III and the MMC cycle length were comparable in the groups. Spontaneous MMC cycles were not disrupted during intracranial hypertension. The mean concentration of lactate and glucose was comparable in the groups, while the concentration of pyruvate was partially higher in the study group than in the controls (p < 0.05). This was associated with a decrease in lactate to pyruvate ratio (p < 0.05).

**Conclusion:**

The present study suggests that a stepwise and hourly increase of the intracranial pressure of up to 50 mmHg, does not influence duodenal motility activity in a significant manner. A considerable deterioration of the duodenal mucosal blood flow was excluded by determining the lactate to pyruvate ratio.

## Background

Raised intracranial pressure (ICP) is described of being associated with a delay in gastric emptying, which may affect toleration of enteral feeding [1,2]. In rats, increased ICP reduced gastric emptying, which seemed to be mediated by the vagus nerve [3]. In patients with a significant brain trauma associated with a decrease of Glasgow Coma Scale level, gastric dysrhythmia was found [4]. Trauma victims with raised ICP also displayed abnormal biphasic response demonstrating a faster gastric emptying during the early stage, while the gastric transit time was prolonged later [2]. Gastric transit time seems to be dominated by pressure pump mechanics resulting from cavity pressure differences between the distal antrum and the proximal duodenum [5]. Thus, duodenal contractile activity might play an essential role in transpyloric transport. While most studies focused on traumatic brain injured subjects in the later phase, there is still a lack of data concerning the initial phase of elevated ICP and its influence on duodenal motility.

The interdigestive motility is normally characterised by periodic occurrence of the migrating motor complex (MMC). The MMC is conventionally divided into three phases: a period of quiescence (phase I) is followed by an irregular contractile activity (phase II), which is replaced by a shorter period of regular contractions termed phase III or the "activity front" of the MMC [6]. To investigate the time period of the interdigestive phases I–III the impedancometry is described as being a reliable tool [7,8], which may provide a more detailed view into the complexity of intestinal functions.

Microdialysis offers opportunities for measuring metabolic changes in the extracellular fluid, which was used as an early indicator of local metabolic deterioration during ischemia [9-12]. Intestinal motor activity can affect mucosal blood flow through passive changes in the vessel caliber due to changes in the vascular transmural pressure [13,14]. In addition, mucosal blood flow has been shown to be reduced during intracranial hypertension [15]. Accordingly, when mucosal ischemia could be excluded as a potential factor of an inhibited intestinal motility, microdialysis could be used to monitor the metabolic changes in the extracellular fluid.

The purpose of our study was to investigate the effects of rising levels of ICP on both the duodenal motility and its microcirculation. Therefore, we applied stepwise and hourly elevated levels of intracranial pressure from its physiological level up to 50 mmHg.

## Methods

The investigation was approved by the local Institutional Animal Ethics Committee. In order to avoid gender bias on intestinal motility we studied eleven male castrated pigs (German Landrace) with a body weight (BW) ranging between 32–40 kg. All animals were obtained from the same breeder. The pigs had free access to water and were fed twice daily with a standard diet (Muskator^®^, Muskator-Werk, Germany).

Each pig was fasted overnight but still had free access to water. After a premedication with 10 mg·kg^-1 ^ketamine i.m. (Ketamin^®^, Cefa Sanre Animale, Germany), general anaesthesia was subsequently induced intravenously with 2–4 mg·kg^-1 ^propofol (Propofol^®^, Parke-Davis, Germany) and further maintained with a continuous infusion of 25 mg·kg^-1^·h^-1 ^propofol and 1–3 μg·kg^-1^·h^-1 ^fentanyl (Fentanyl-Janssen^®^, Janssen-Cilag, Germany). The dosages of propofol and fentanyl were guided by clinical signs to ensure appropriate depth of anaesthesia. The pigs underwent orotracheal intubation and were mechanically ventilated. Oxygen saturation (SaO_2_) of between 94–99%, and expiratory carbon dioxide partial pressures (p_ET_CO_2_) of between 35–38 mmHg were maintained. Fluid was replaced during surgery with a crystalloid solution at a rate of 2 ml·kg^-1^·h. The blood pressure was measured through invasive (A. femoralis using Leader Cath^®^, Vygon, France) and non-invasive methods on the hind limb (Riva-Rocci; AS/3 Compact^®^, Datex-Engstrom, Finnland and Dura Cuff™, Infant Cuff, Criticon, USA). Mean arterial blood pressures (MAP), heart rate (HR), SaO_2_, and p_ET_CO_2 _were assessed at 15 minute intervals during the surgical procedure. The body temperature (Temp) was measured twice a day as well as the white blood cell count (WBC).

The animals were instrumented with a central venous catheter (CVC) inserted into the left jugular vein via a venous cut down. Pigs underwent laparotomy and both an impedance catheter (2 mm diameter, 100 cm length, flexible, 14 electrodes distributed over the distal 28 cm, Femu-Research Institute, Germany) and a catheter for microdialysis (CMA/20^®^, membrane length 10 mm, cut-off 20000 Dalton, CMA/Microdialysis, Sweden) were manually introduced into the duodenum before the duodenum and the abdominal wall were sutured.

All animals were instrumented with a ventricular catheter which was inserted into the left frontal horn, at a depth of 20–35 mm from the cortical surface. The catheter was connected with a pressure transducer (Pressure Set, Smith Medical Deutschland GmbH, Germany). The same canal was used to apply raising levels of ICP by a bolus and subsequently continuous infusion of an artificial cerebrospinal fluid (Ringer Solution + 100 mmol/l glucose) into the ventricular system via a three-way-tap. In addition to the physiological state, the ICP was adjusted to 10, 20, 30, 40, and 50 mmHg in pigs in the verum group. Every level of the ICP was retained for one hour and was controlled intermittently via pressure transducer (AS/3 Compact^®^, Datex-Engstrom, Finnland). In contrast, pigs during physiological state without any ICP adjustments served as the control group.

**Figure 1:**
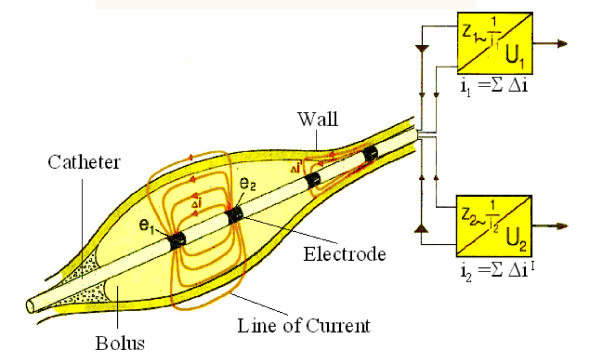
Cylinder shaped metallic electrodes (Electrode) are mounted on a thin plastic catheter, which was intraluminal introduced (Wall). Each neighboring electrode pair is connected to an impedance voltage transducer outside the body (Zn = measured impedance of an electric field between two electrodes at the moment, which is the ratio between the applied voltage (U) and the resulting current (I). The instantaneous output voltage of each transducer represents the average electrical impedance of the volume conductor around the catheter.

**Figure 2:**
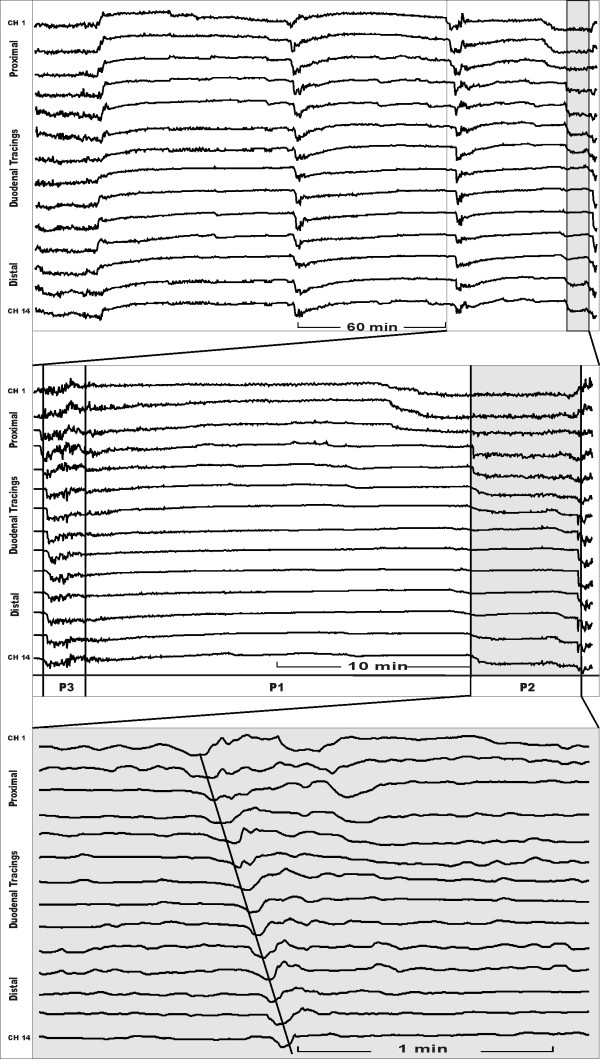
Impedance tracings of a pig during an elevated ICP period. Tracings demonstrate 14 pairs of electrodes (Ch 1 = proximal channel 1; Ch 14 = distal channel). Top: time period of 4 hours demonstrating interdigestive phases. Middle: complete MMC cycle distracted, p1 = phase I, p2 = phase II, p3 = phase III. Bottom: phase II further expanded demonstrating a bolus transport event (BTE).

Duodenal motility was assessed by impedancometry (Figure 1). Locally developed software (Femu-Research Institute, Germany) was used for the data acquisition and analysis. According to established manometric criteria the MMC was defined as the time interval between two successive phase III patterns. Each interdigestive phase I–III was analysed according to the criteria described by Malagelada et al. [6]. The following parameters were investigated: Duration of duodenal interdigestive phases I–III and MMC cycle length, and the quantity of duodenal bolus transport events (BTE). Duodenal motor patterns and BTE were defined as described by Nguyen et al. [7] (Figure 2). The BTE was defined as particular impedance tracing which is associated with a passage of a bolus detected over three and more measuring channels (Figure 2).

The inserted microdialysis catheter was connected with low-volume FEP-tubing (1.2 μl/10 cm) to a precision infusion pump (CMA 102, CMA Microdialysis, Sweden) in order to maintain a constant dialysate flow. The probe was continuously perfused at a flow rate of 2 μl/min with a solution containing 147 mmol/l sodium, 156 mmol/l chloride, 4 mmol/l potassium, 2.3 mmol/l calcium, (Perfusion fluid T1, CMA Microdialysis, Sweden). After an equilibration time of 60 minutes, dialysate samples were continuously collected in 20 min intervals and were immediately frozen at -20°C until analysed. The day after the experiment, defrozen and centrifuged dialysate samples were analysed enzymatically with a CMA 600 Microdialysis Analyser (CMA/Microdialysis, Sweden) for lactate, pyruvate, and glucose. The exchange of substances across the microdialysis membrane is limited by the total area of the membrane, the perfusion flow rate, the characteristics of the diffusing substance and the diffusion constant in the tissue surrounding the probe [12,16]. The recovery rate expresses the relation between the concentration of the substance in the microdialysis probe effluent and the concentration of the medium [17]. At the end of the experiment, the microdialysis probes were removed from the duodenum and the recovery rates for each probe were determined by proceeding with the perfusion and the same settings in a calibrated solution. Concentrations of the calibrated solutions were compared with the concentrations of the in vitro microdialysis samples to determine the relative recovery for each substance. Measured experimental values were weighted with the relative recovery to estimate the in vivo extracellular concentration of the components in the immediate vicinity of the probes. In vitro recovery rates were 42% ± 6 for lactate, 44% ± 9 for pyruvate, and 28% ± 6 for glucose.

In order to ensure standard experimental conditions the following parameters were monitored: Heart rate, mean arterial blood pressure, body temperature, white blood cell count, arterial blood gas analysis. At the end of the measurements, the animals were euthanised with an overdose of barbiturate.

### Statistical analysis

Microdialysate data of each group was analysed using the Kruskal-Wallis Test. Where differences occurred, data of the individual time points were compared by using the Bonferroni corrected Wilcoxon Rank-Sum Test. Differences of the length of motility phases between the groups were analysed using the Mann-Whitney U test. Statistical analysis was performed using statistical software (NCSS^®^, NCSS Statistical Software, USA). Data are expressed as median and range or mean and standard error of the mean (SEM) when necessary. P-values less than 0.05 were considered significant.

## Results

**Figure 3:**
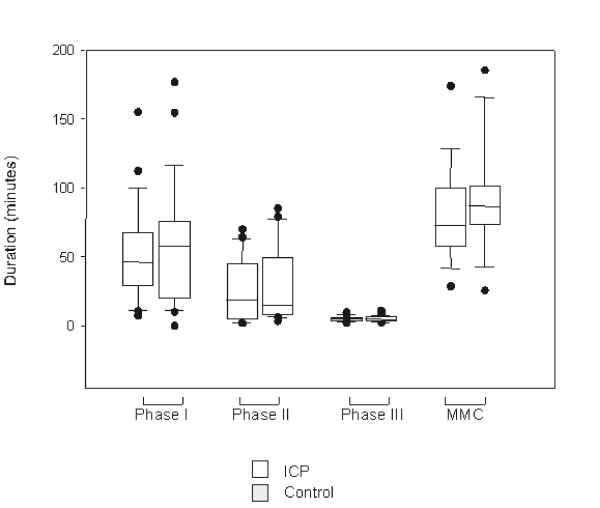
Duration of interdigestive phases I–III and MMC cycle length of verum and controls. Data are shown as box blots indicating median, 25% and 75% quartiles, and range.

All motility phases showed a comparable length between the groups. Accordingly, the MMC cycle length did not differ between the pigs with increased ICP's and the control. Spontaneous MMC cycle did not seem to be disrupted during the elevation of the ICP. The median duration of the interdigestive phases I–III is demonstrated in Figure 3. All BTE's analysed have been characterised as long distance and propulsive BTE. The median quantity of BTE during each phase is shown in Table 1, which demonstrates a comparable BTE count between the groups.

**Table 1:** Number of bolus transport events (BTE) during the interdigestive phases I–III expressed as median and range.

	**Control**	**ICP**	**p**
phase I	2.5 (0–15)	3 (0–43)	0.3
phase II	2 (0–30)	3.5 (0–28)	0.3
phase III	0 (0–18)	0 (0–15)	0.1

**Figure 4:**
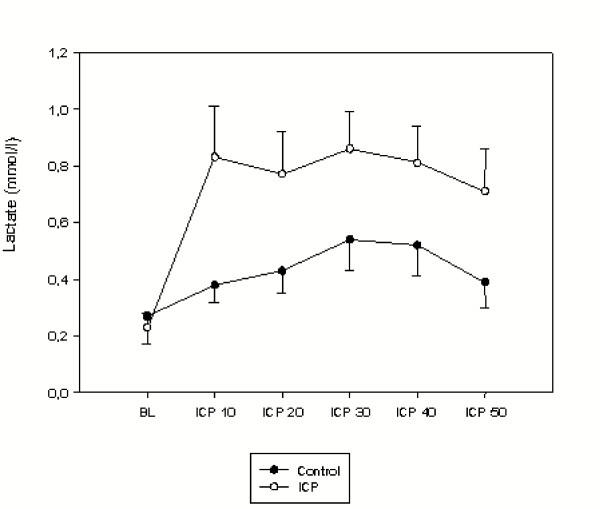
Intraluminal lactate concentration (mmol/L) during baseline (BL) and increasing levels of intracranial pressures (ICP10 = ICP of 10 mmHg). Data are expressed as mean ± SEM.

**Figure 5:**
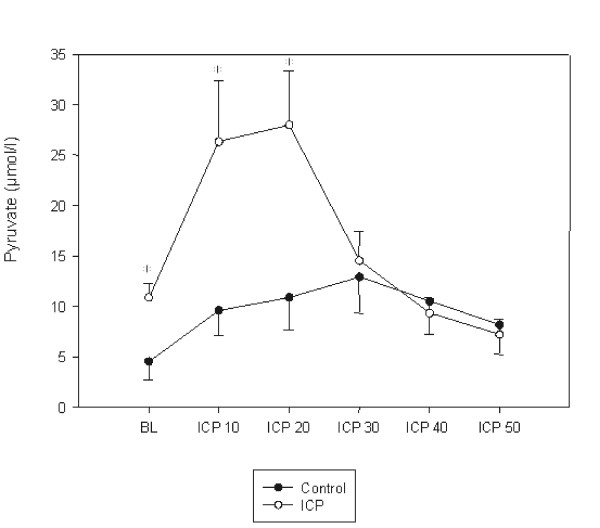
Intraluminal pyruvate concentration (μmol/L) during baseline (BL) and increasing levels of intracranial pressures (ICP10 = ICP of 10 mmHg). Data are expressed as mean ± SEM. Significance between verum and controls is defined as (*) when p < 0.05.

**Figure 6:**
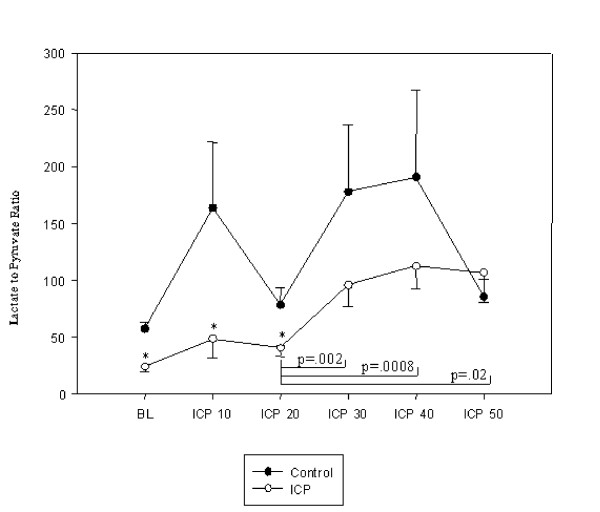
Lactate to pyruvate ratio during baseline (BL) and increasing levels of intracranial pressures (ICP10 = ICP of 10 mmHg). Data are expressed as mean ± SEM. Significance between verum and controls is defined as (*) when p < 0.05.

**Figure 7:**
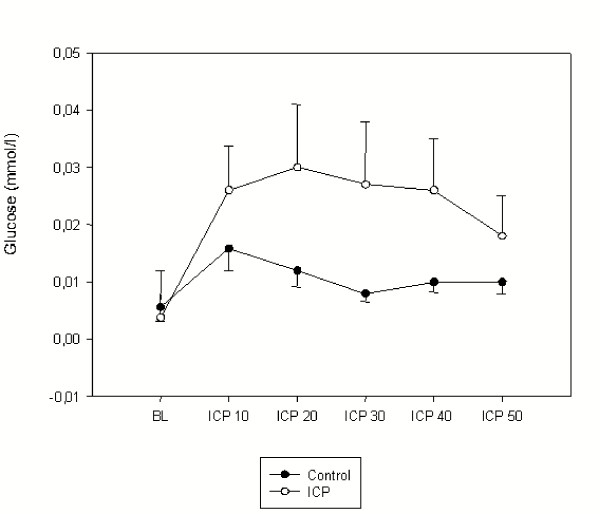
Intraluminal glucose concentration (mmol/L) during baseline (BL) and increasing levels of intracranial pressures (ICP10 = ICP of 10 mmHg). Data are expressed as mean ± SEM.

The mean concentrations of lactate and glucose were comparable in the groups. During baseline the mean concentration of pyruvate was higher in the group with elevated ICP than in the controls (p < 0.05), hence this was associated with a decrease in the lactate to pyruvate ratio (p < 0.05). The mean concentrations of lactate, pyruvate, glucose, and the lactate to pyruvate ratio are presented in Figures 4, 5, 6, 7. During the elevation of the ICP, there was a trend towards increased levels of lactate and glucose, without showing any statistical significance. Pyruvate concentrations were increased during a raise in the ICP from 10–20 mmHg (p < 0.05), which decreased again during further elevations of the ICP of up to 50 mmHg. This initial increase in pyruvate was associated with a decreased lactate to pyruvate ratio (p < 0.05). During further elevation of the ICP from 20–50 mmHg, the lactate to pyruvate ratio was similar in the groups. Simultaneously, an increase in the lactate to pyruvate ratio (p < 0.05) was measured, when compared to the controls.

All animals were included into the study. Different levels of ICP were continuously adjusted without any influence on haemodynamics. Values of mean arterial blood pressures and heart rates did not differ between the groups (Table 2). There were no differences in median blood glucose level (5.3 mmol/L (range: 4.2–7.4 mmol/L)) or median blood lactate level (0.9 mmol/L (range: 0.59–1.87 mmol/L)) detectable between the groups. The pH was comparable in the groups with a median of 7.52 (range: 7.42–7.6), as were the pO_2 _(median: 161.5 mmHg [range: 75.3–178.8 mmHg]) and the pCO_2 _(median: 32.7 mmHg [range: 25.1–41.3]).

**Table 2:** Biometric and plasmatic data of the study group (ICP) and controls presented in median and range. Statistical significance is defined when p < 0.05. MAP = mean arterial blood pressure at baseline (MAP-BL) and during intracranial pressure (MAP-ICP) between 10–50 mmHg including corresponding MAP of controls (Control); SaO_2 _= oxygen saturation; WBC = white cell blood count

	**Control**	**ICP**	**p**
Heart rate (/min)	83 (62–110)	83 (62–110)	0.3
MAP-BL (mmHg)	97 (80–102)	87 (65–112)	0.6
MAP-ICP 10 (mmHg)	97 (89–108)	87 (65–112)	0.3
MAP-ICP 20 (mmHg)	92 (67–103)	81 (59–127)	0.7
MAP-ICP 30 (mmHg)	98 (82–106)	84 (59–127)	0.1
MAP-ICP 40 (mmHg)	92 (67–116)	74 (59–116)	0.1
MAP-ICP 50 (mmHg)	93 (84–105)	75(57–121)	0.1
SaO_2 _(%)	99 (98–100)	98 (96–100)	0.3
WBC (×10^3^/μl)	13.4 (12.3–17.8)	13.6 (10.7–24.2)	0.7
Temperature (°C)	35.8 (32.6–37.8)	35.3 (34.2–38.5)	0.2
Hematocrit (%)	29 (21–33)	29 (26–31)	0.1
Lactate (mmol/l)	0.77 (0.59–1.76)	0.97 (0.66–1.87)	0.2

## Discussion

The present study suggests that an acute and hourly increase of the intracranial pressure up to 50 mmHg did neither influence the spontaneous duodenal MMC cycle length nor the sequence or time periods of the interdigestive phase I–III of the proximal duodenum. A considerable deterioration of the mucosal blood flow can be excluded by the lactate to pyruvate ratio.

The spontaneous MMC cycle length has been demonstrated to last 75–80 minutes, characterised by myoelectrography in the fasted pig [18]. In the present study, the median MMC cycle lengths in the controls and verum were 87 and 73 minutes, respectively, demonstrating a trend towards a slightly shortened MMC cycle length in pigs with raised ICP. This seems to be due to a slightly and non-significant shortened phase I, so called the phase of quiescence. Usually, phase I activity demonstrates minimal aboral propulsion of luminal contents and occupies 40% to 60% of the overall MMC cycle time [19]. A trend towards a shortened phase I leading to a shortened MMC cycle length could suggest a faster intestinal transit time which could help to explain the increase in gastric emptying during the first days after the head trauma described by Ott et al. [2]. Aboral food migration seems to depend on duodenal propulsive motility activity, which is normally of antral origin and propels food aboral through the small intestine [20]. It is suggested that transpyloric flow is determined through active and co-ordinated processes involving not only antral contraction but also duodenal motility and pyloric function. In pigs, gastric emptying has been shown to predominantly occur during the non-lumen-occlusive stage of the propagated gastric contractions with emptying rates being directly related to the rate of antropyloric pressure waves and being inversely related to the rate of isolated pyloric pressure waves [21,22]. In humans, transpyloric flow has been shown to be increased during the usually occurring cavity pressure difference between the distal antrum and the proximal duodenum at periods that demonstrate quiescence in gastric contractions [5]. Therefore, a shortened phase I might also help to explain a prolonged transpyloric transit time probably caused by an abbreviated phase of quiescence, this being associated with a reduction in the antroduodenal pressure difference.

Phase II demonstrates increasing contractile activity which has been characterised by being mainly involved in peristaltic mechanisms responsible for the transpyloric gastroduodenal flow [23]. A bolus transport event (BTE) is described as a particular impedance tracing related to a bolus passage [7]. This aspect can be confirmed by our findings of concomitant BTE's during phase II. In contrast, BTE's which were found during phase I are much more difficult to explain, because phase I has been defined as a phase of quiescence and this normally does not demonstrate any significant coordinated food migration [8]. In order to further elucidate the function of these BTE's occurring during phase I as well as the length of time of phase I and its role in transpyloric transport, the impedance technique should therefore be used in conjunction with real-time ultrasound and manometry.

In total, the present non-traumatic porcine model using a short-term and stepwise increase of the ICP did neither disturb spontaneous MMC cycles nor affect the quantity of BTE. This might be due to the investigated duration of raised ICP (20–50 mmHg) over 4 hours that might be too short to initiate a fastened gastrointestinal transit described during the early phase of intracranial hypertension. This aspect might be supported by the findings of Ott and co-workers [2] who found that significant changes in gastric emptying happened 1 to 2 weeks after the injury. On the other hand, the fastened gastric emptying after head trauma could be an isolated gastric phenomenon that can not be detected by duodenal impedancometry. Garrick et al. [24] found that gastric and duodenal contractile forces were reduced reversibly by short term elevated intracranial pressure of up to 13 cm H_2_O in the conscious rabbit. The lack of significant effects during raised ICP in the present study might be, at least partially, explained by the fact that vagal tone in anaesthetised animals is considerably lower than in the unanaesthetised subjects. In order to accurately define duration of the interdigestive phase I–III during a more extended phase of elevated ICP further studies are necessary.

In the present study, pigs with 32 to 40 kg body weights were investigated because previous experiences allowed us to use devices comparable to those usually used in humans [25,8]. In general, the pig is considered to be a suitable non-primate animal model since it resembles the human situation with regard to eating behaviors, anatomy, and physiology of the gastrointestinal tract [26]. Clinical studies demonstrated that the luminal impedance technique was a reliable tool to investigate motility activities [20,27]. The impedance technique allows the gastroduodenal motility patterns to be determined by the acquisition of electrical impedance in the surrounding body volume conductor from a number of annular electrodes [28]. While the passage of air or gas results in a temporary increase in intraluminal impedance, the passage of hyperconductive fluid is followed by a decrease in impedance. Thus, the impedance technique describes gastroduodenal fluid transport independent of associated luminal pressure events and, therefore, might be more precise than manometry. Although, the BTE's have been previously defined as propulsive activities being associated with the transport of bowel contents [7], intestinal motility detected by the single use of the impedance technique can not be directly linked with luminal pressure activity. This aspect has to be taken into account when our results are to be compared with those recorded by using the manometry.

Furthermore, the use of luminal microdialysis in monitoring intestinal ischemia has already been reported [10,11]. In the present study there was a trend of slightly decreased mean arterial blood pressure during elevated ICP without statistical significance. This was paralleled by a trend of an initial increase of luminal lactate concentrations when ICP was elevated from baseline to 10 mmHg, while we could not find any significant differences when compared to the controls. Simultaneously, luminal increase of pyruvate resulted in a significant decrease of the luminal lactate to pyruvate ratio during the elevated ICP period indicating a lack in any deterioration of the duodenal blood supply. Accordingly, glucose concentrations seemed to be unaffected during the time period of the elevated ICP. This finding led to the assumption that an acute and short time elevation of ICP did not reduce mucosal blood flow in any significant manner.

We preferred the luminal approach, because, in contrast to the intestinal wall, the intraluminal insertion of the microdialysis catheter seems to be easier to perform especially when tissue trauma and destruction of the catheter itself should be avoided. Yet, it remains unclear whether a more extended duration of raised ICP would have influenced the mucosal blood supply in this study. Data concerning the time period of intracranial hypertension and its influence on mucosal blood flow are lacking. In the present study haemodynamics were stable and comparable between the groups. Accordingly, dosages of propofol and fentanyl that were guided by clinical signs to ensure appropriate depth of anaesthesia were also comparable, which is of great importance since both propofol and fentanyl demonstrate a potential inhibitory effect on gastrointestinal motility [29,30]. For the future, stable haemodynamics might be of great importance, since hypoperfusion of the small intestine might be associated with impaired gut barrier function, which is thought to have a high impact on the development of multiple organ failure [31,32], and the long-term outcome of severely brain injured patients.

## Conclusion

The present study suggests that short-term elevated levels of intracranial pressures neither show any significant influence on the duodenal motility activity nor decrease in duodenal mucosal blood flow.

## Competing interests

The author(s) declare that they have no competing interests.

## Authors' contributions

JoS made substantial contributions to the conception, design, and interpretation of the collected data. He performed the study and drafted the manuscript. NZ and BK performed the study and provided critical review of the manuscript. JiS and RR contributed to the design of the study and the interpretation of the data and provided critical review of the manuscript. All authors read and approved the manuscript.

## Pre-publication history

The pre-publication history for this paper can be accessed here:


